# Diagnostic utility of DNA methylation episignature analysis for early diagnosis of KMT2B-related disorders: case report

**DOI:** 10.3389/fgene.2024.1346044

**Published:** 2024-02-15

**Authors:** Nadia Bouhamdani, Haley McConkey, Amélie Leblanc, Bekim Sadikovic, Mouna Ben Amor

**Affiliations:** ^1^ Vitalité Health Network, Moncton, NB, Canada; ^2^ Faculty of medicine and health sciences, Université de Sherbrooke, Sherbrooke, QC, Canada; ^3^ Centre de Formation Médicale du Nouveau-Brunswick, Université de Moncton, Moncton, NB, Canada; ^4^ Department of Chemistry and Biochemistry, Université de Moncton, Moncton, NB, Canada; ^5^ Verspeeten Clinical Genome Centre, London Health Sciences Centre, London, ON, Canada; ^6^ Department of Pathology and Laboratory Medicine, Schulich School of Medicine and Dentistry Western University, London, ON, Canada

**Keywords:** Kmt2b, dystonia, DYT28, case report, childhood dystonia, deep brain stimulation, epigenetic, episignature

## Abstract

The lysine methyltransferase 2B (KMT2B) gene product is important for epigenetic modifications associated with active gene transcription in normal development and in maintaining proper neural function. Pathogenic variants in KMT2B have been associated with childhood-onset Dystonia-28 and Intellectual developmental disorder, autosomal dominant 68 (MRD 68) for cases of neurodevelopmental impairment without dystonia (DYT28; OMIM 617284 and MRD68; OMIM 619934, respectively). Since its first description in 2016, approximately one hundred KMT2B genetic variants have been reported with heterogeneous phenotypes, including atypical patterns of dystonia evolution and non-dystonic neurodevelopmental phenotypes. KMT2B-related disorders share many overlapping phenotypic characteristics with other neurodevelopmental disorders and delayed dystonia, that can appear later in childhood, often delaying clinical diagnosis. Furthermore, conventional genetic testing may not always provide actionable information (e.g., gene panel selection based on early clinical presentation or variants of uncertain significance), which prevents patients and families from obtaining early access to treatments and support. Herein, we describe the early diagnosis of KMT2B-related neurodevelopmental disorder by DNA methylation episignature testing in a 4-year-old patient without features of dystonia at diagnosis, which is reported to develop in more than 80% of KMT2B-related disorder cases. The proband, a 4-year-old female of Jewish-Israeli descent, presented with speech delay, microcephaly, poor weight gain, attention-deficit and hyperactivity disorder, dysmorphism, intellectual disabilities and joint hyperlaxity, but presented no signs of dystonia at initial evaluation. Episignature screening in this pre-symptomatic patient enabled accurate genetic diagnosis and timely and actionable intervention earlier in the natural history of Childhood-onset Dystonia-28.

## 1 Introduction

Dystonia is a movement disorder characterized by uncontrolled repetitive movements or postures due to continuous or intermittent muscle contractions and its early onset often leads to severe disability in children (Li et al., 2020; Smit et al., 2021). Heterozygous variants in the KMT2B gene, encoding a histone H3 methyltransferase, have been linked to childhood-onset Dystonia 28 and Intellectual developmental disorder, autosomal dominant 68 (MRD68) for cases of neurodevelopmental impairment without dystonia (Zech et al., 2016; Meyer et al., 2017; Zech et al., 2019). KMT2B-related dystonia (also known as DYT28, DYT-KMT2B, Dystonia 28) is an autosomal dominant complex childhood-onset movement disorder and is emerging as an important and frequent cause of progressive generalized dystonia (Gorman et al., 2018). With a median age at symptom onset of 7.0 years, the disease course commonly evolves from lower-limb focal dystonia resulting in foot deformity, toe walking, or gait disturbances, into generalized dystonia with prominent cervical, cranial, and laryngeal involvement, resulting in dysphagia and/or dysphonia (del Toro-Pérez and Romero, 2022; Abela and Kurian, 2018). Unlike other dystonias, pathogenic variants in KMT2B have also been associated with additional features such as facial dysmorphia (e.g., elongated face with nasal tip) as well as intellectual disability and preceding developmental delay in more than 50% of reported cases (del Toro-Pérez and Romero, 2022).

Since the first description of KMT2B-related dystonia in 2016, novel disease presentations and heterogeneous phenotypes are being described such as atypical patterns of dystonia evolution and non-dystonic neurodevelopmental phenotypes, as well as varying age of symptom onset (Zech et al., 2016; Zech et al., 2019; del Toro-Pérez and Romero, 2022; Cif et al., 2020). The growing phenotypic variability and broad clinical spectrum have not only presented a diagnostic challenge but have added an additional difficulty for physicians in assuring an early diagnosis of this rare disease (Cif et al., 2020). Further complicating the differential diagnosis, a distinct subtype of KMT2B patients present with a neurodevelopmental phenotype in the absence of dystonia (MRD68). In addition to the broad clinical spectrum of this disease, KMT2B haploinsufficiency has been implicated with more than one hundred single nucleotide variants or insertions/deletions, with rare missense variants being associated with generalized dystonia (Zech et al., 2016; Meyer et al., 2017; Zech et al., 2017; Carecchio et al., 2019; Cif et al., 2020). Furthermore, variants of uncertain significance and complex inheritance patterns add complexity to the interpretation of genetic findings (Abela and Kurian, 2018).

Herein, we demonstrate the utility of DNA methylation episignature testing for the early diagnosis of KMT2B-related neurodevelopmental disorder in a 4-year-old female not displaying dystonia at initial evaluation, however this symptom can develop within the course of the disease, and has been reported in the majority of KMT2B-related disorder cases, allowing for early patient-tailored care, treatment recommendations, and surveillance. The methylation profile identified for this patient matched the Dystonia 28 episignature; all samples used to develop the episignature displayed dystonia (Ciolfi et al., 2021). The patient presented with a non-specific phenotype, demonstrating speech delay, microcephaly, poor weight gain, attention-deficit and hyperactivity disorder, dysmorphism, concerns about intellectual abilities and joint hyperlaxity. Among the large list of differential diagnoses, KMT2B-related dystonia 28 was considered as a less likely possibility given the patient was not yet displaying dystonia and their other clinical presentation overlapped with many other neurodevelopmental disorders. Episignature biomarkers provide a powerful tool for timely diagnosis of rare diseases, such as KMT2B-related Dystonia 28, whose incidence, genetic variability, and clinical spectrum are not completely defined.

## 2 Case presentation

A 4-year-old patient of Jewish-Israeli descent, without known consanguinity, was evaluated in the Medical Genetics Clinic upon referral from a pediatrician regarding speech delay (no words until the age of 3.5 years) and microcephaly. The patient who is a product of spontaneous conception, as well as an uncomplicated pregnancy with no history of maternal illness or exposure to teratogens, was delivered at 39 weeks by induced vaginal delivery for intrauterine growth restriction diagnosed in the third trimester, prompting an amniocentesis that came back normal. There was no explanation determined for intrauterine growth restriction despite routine investigations. The father is a 37-year-old male with no known health issues and the mother is a 34-year-old female with anemia. The parents’ head circumferences also ranged within normal limits. The patient is a sibling of a healthy 9-year-old girl, a 7-year-old girl who struggled with speech delay that has since resolved and a healthy 3-year-old boy. The patient was previously diagnosed with early onset attention deficit hyperactivity disorder (ADHD) by snap IV questionnaire, but no medication was indicated at that time.

Upon physical examination, the patient’s weight was at 14.8 kg, at the 10th percentile, height at 108.3 cm, at the 50th percentile, and head circumference was measured at 45.5 cm at more than 3.5 standard deviations below the mean. No motor delays, regression in skills, or sensory issues were noted. The musculoskeletal exam revealed joint hyperlaxity. Bilateral clinodactyly of the 5^th^ finger and partial bilateral skin syndactyly with overlapping toes were highlighted. Dysmorphic facial features were present; the patient presented with bitemporal narrowing, epicanthal folds and long eyelashes, depressed nasal bridge, flat smooth philtrum and a large mouth. There was also a horizontal fissure on the right ear lobe and abnormal hair whorls distribution. Cardiopulmonary, abdominal, genital, and neurological exams were normal. The extremity and axial tone as well as the reflexes were also normal. Finally, the tegument exam revealed no abnormal skin pigmentation. Concerns regarding intellectual abilities were also noted.

### 2.1 Investigation

Previous investigations preceding the initial evaluation at the Medical Genetics Clinic included chromosomal microarray, that did not reveal any clinically relevant genomic deletions or duplications. Absence of heterozygosity was seen at multiple chromosomal locations, representing a minimum of 3.9% of the patient’s autosomal genome, which does not on its own represent an abnormal result. The differential diagnosis included syndromic microcephaly, autosomal recessive primary microcephaly, metabolic conditions including aminoaciduria/organic acidurias, creatine deficiency disorders, urea cycle disorders, Smith-Lemli-Optiz syndrome, thalassemia, Angelman syndrome, and DNA repair defects. DNA repair defects as the cause of the proband’s phenotype, however, was unlikely in the absence of a short stature or susceptibility to infections. Results from all molecular tests came back normal. Specifically, methylation patterns for Angelman syndrome were normal, urine creatine was normal, the urine organic acid and amino acid profiles did not suggest a metabolism disorder, sterols profile was normal and complete blood count results did not suggest a diagnosis of thalassemia. The patient met the criteria for enrollment in the “EpiSign-CAN: Beyond Genomics: Assessing the Improvement in Diagnosis of Rare Diseases using Clinical Epigenomics in Canada” study, and a request to consent and enroll the patient occurred at same time as other first-tier tests (metabolic work up and methylation analysis for Angelman syndrome) were ordered. This Canadian national study involves 16 participating Genetics Centres and aims to assess the diagnostic and clinical utility of the first clinically validated test able to analyze genomic DNA methylation for diagnosis of rare genetic diseases. The EpiSign™ test involves screening of a growing number of DNA methylation episignature biomarkers associated with rare disorders. EpiSign™ technology uses machine learning derived algorithms to compare a patient’s DNA methylation profile to established episignatures within the EpiSign Knowledge Database, which houses thousands of reference methylation profiles for hundreds of rare diseases (Haghshenas et al., 2020; Sadikovic et al., 2021; Foroutan et al., 2022; Kerkhof et al., 2022; Levy et al., 2022; McConkey et al., 2022). The patient’s family was available to provide consent to participate in the EpiSign-CAN study a month after the initial evaluation, with the intent of using EpiSign™ as part of first-tier testing.

### 2.2 Results

**FIGURE 1:**
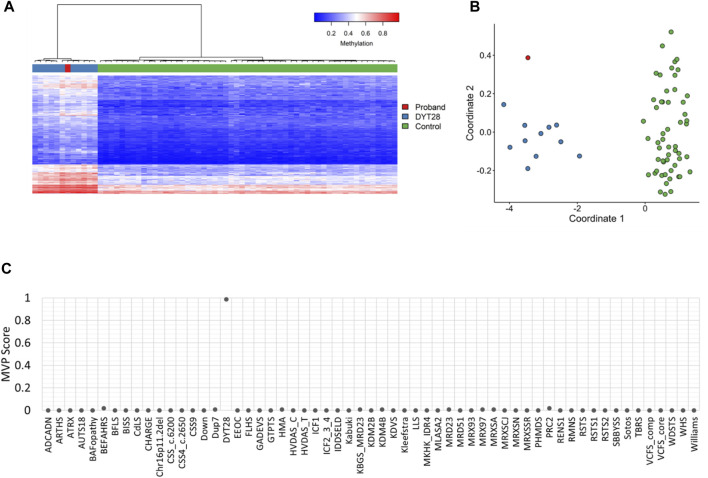
EpiSign™ DNA methylation analysis of peripheral blood from a proband. **(A)** Hierarchical clustering and **(B)** multidimensional scaling plots indicate that the patient (red) has a DNA methylation signature similar to subjects with a confirmed Dystonia 28 (DYT28) episignature (blue) and distinct from controls (green). Each row of the heatmap represents one CpG probe on the DNA methylation array, and each column represents one individual’s sample. The heatmap color scale from blue to red represents the DNA methylation level (beta value) from 0 (no methylation) to 1 (fully methylated). **(C)** MVP score, a multiclass supervised classification system capable of discerning between multiple episignatures by generating a probability score for each episignature. The elevated patient MVP score for DYT28 compared to other syndromes suggests an episignature concordant with the DYT28 reference signature.

EpiSign™ analysis was perfomed at London Health Sciences Centre in London, Ontario. Assessment of patient DNA extracted from peripheral blood revealed an episignature consistent with Dystonia 28, despite no signs of dystonia in the patient at the time of evaluation. The episignature detected for this patient was concordant with the methylation profile observed in patients with Dystonia 28, Childhood-onset syndrome, including Euclidean clustering (Figure 1A) and multidimensional scaling (Figure 1B) consistent with Dystonia 28-specific methylation changes. Additionally, the Methylation Variant Pathogenicity (MVP) score for this patient was positive for Dystonia 28, resulting in a conclusive high-confidence positive result for the Dystonia 28 methylation signature (Figure 1C). This result was concordant with the follow-up KMT2B single gene sequencing, with deletion and duplication analysis, in a clinical accredited laboratory, which confirmed a *de novo* heterozygous pathogenic variant in KMT2B: c.12_24dup (p.Ser9Glyfs*111) (ClinVar: RCV000991204.1, RCV000521909.1).

### 2.3 Follow-up and recommendations

**FIGURE 2:**
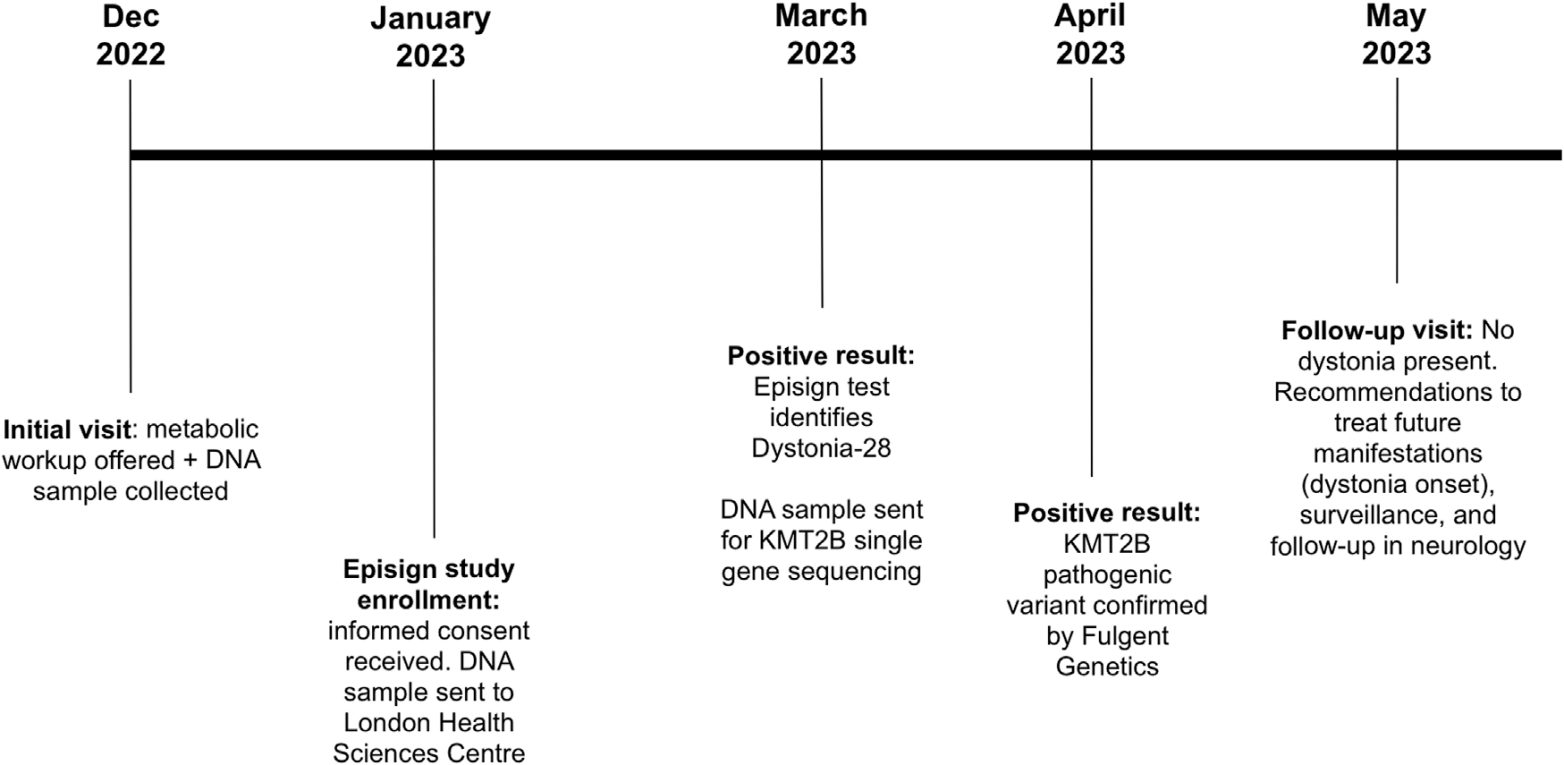
Timeline of the patient being followed at the Medical Genetics Clinic in New Brunswick. Patient’s initial visit consisted in offering a metabolic workup and a DNA sample was collected for future investigations. Patient was then enrolled in the Episign study. Results came back positive with identification of Dystonia-28. KMT2B single gene sequencing was thus requested and as expected, KMT2B pathogenic variant was confirmed. At the time of the follow-up visit, no dystonia was present in the patient. Recommendations were given to treat possible future manifestations of dystonia onset as well as surveillance, and a follow-up in neurology.

During the most recent follow-up appointment, the patient, who was now 5 years old, had experienced three episodes of seizures since the initial evaluation (Figure 2). As a result, the proband was diagnosed with recent onset partial complex seizures based on an abnormal Electroencephalogram that showed epileptiform discharge in the right posterior head region with persistent focality which raised concerns of an underlying structural abnormality. She was started on Trileptal, 150 mg PO BID. At this follow-up evaluation, the patient still did not present dystonic features and did not yet present spasticity or myoclonus; however, during the physical exam, an abnormal gait with slight tendency for toe walking and stiffness was noticed on the patient’s right lower extremity (Figure 2).

The patient was referred to a pediatric neurologist for a complete detailed neurologic examination and for surveillance and follow-ups in the event of later dystonia onset (Figure 2). If this is the case, an early assessment for possible effectiveness of deep brain stimulation (DBS) will be highly beneficial. Physiotherapy, occupational therapy, and speech and language therapy assessments were also recommended. Surveillance will include regular monitoring of the following: onset of dystonic features, nutritional status, swallowing to evaluate risk for aspiration, speech and language regarding needs for augmentative communication, adaptive functioning, potential orthopedic complications (specifically hip dislocation and kyphoscoliosis with hip and spine x-rays every 6–12 months), psychiatric status, and skin examination for changes requiring appropriate management.

## 3 Discussion

We report the early diagnosis of KMT2B-related neurodevelopmental disorder in a 4-year-old patient who presented no dystonic features at the time of referral and initial evaluation at the Medical Genetics Clinic. EpiSign™ testing allowed early diagnosis of this rare genetic disorder, shortly after the patient’s initial evaluation, by matching her DNA methylation profile with the established episignature for Childhood-onset Dystonia 28. The detected episignature was later confirmed by targeted single gene sequencing of KMT2B in an accredited laboratory. A *de novo* pathogenic heterozygous c.12_24dup (p.Ser9Glyfs*111) variant in *KMT2B* was confirmed after trio testing. This pathogenic variant is predicted to result in a frameshift in exon 1 which introduces a premature stop codon at least 50 nucleotides upstream of the canonical donor splice site of the penultimate exon. This is predicted to result in the loss of function of the protein due to nonsense-mediated mRNA decay.

The detected episignature for this patient was developed using dystonic patients with *bona fide* pathogenic variants in *KMT2B* (Ciolfi et al., 2021). This episignature was tested on 10 patients with variants of uncertain significance (VUS), with only 2 matching the episignature. Both of these patients displayed dystonia. Of the remaining 8 patients with a VUS who did not match the episignature, 5 displayed dystonia, 2 did not display dystonia and 1 only displayed dystonia during paroxysmal attacks (Ciolfi et al., 2021). While all patients who matched the episignature presented with dystonia, testing of samples from patients with pathogenic variants in *KMT2B* but who do not display dystonia (and are older than the age range of onset) has not been completed. Therefore, we cannot state whether this episignature is specific to the dystonia phenotype, and perhaps there would be two different KMT2B episignatures, one that represents the dystonic phenotype and another that detects non-dystonic patients, or rather it is the functional consequence of loss or abnormal function of KMT2B despite resulting presentation. Other groups have worked on and developed methylation profiles for KMT2B. One group compared methylation changes between patients with KMT2B-related dystonia and patients with KMT2D-related kabuki syndrome (Lee et al., 2022). The authors found distinct methylation changes associated with each lysine methyltransferase but that the majority of methylation changes were hypermethylation for both disorders. They also tested and confirmed the episignature developed and used for this patient’s case as well as another episignature developed by Mirza-Schreiber *et al* (Mirza-Schreiber et al., 2022)*.* All groups found predominant hypermethylation associated with KMT2B deficiency (Ciolfi et al., 2021; Lee et al., 2022; Mirza-Schreiber et al., 2022). Finally, Monfrini et al., 2022 investigated differing DNA methylation profiles between *KMT2B-*related dystonia patients based on dystonia onset. This group found that methylation profiling was able to distinguish adult-onset patients from both early onset patients and controls, suggesting that specific missense variants in *KMT2B* may act as genetic determinant of adult-onset of dystonia.

Approximately one hundred KMT2B variants have been reported to date with a wide spectrum of phenotypes, including dystonic and non-dystonic symptoms (Zech et al., 2016; Zech et al., 2019; del Toro-Pérez and Romero, 2022; Cif et al., 2020). KMT2B-related disorders are associated with variable, complex, and often non-specific clinical features that overlap with other neurodevelopmental diseases, making a definitive and early diagnosis challenging (Sadikovic et al., 2021; Smit et al., 2021). This is well illustrated in a recent study which describes the clinical and genetic features of the largest cohort of KMT2B-related disorder patients reported to date (*n* = 53 with an additional analysis of 80 published cases) (Cif et al., 2020). Interestingly, 93% of the patient cohort presented with additional neurological, psychiatric, and non-neurological systemic features, suggesting that most had a complex dystonic phenotype. Furthermore, several KMT2B-related Dystonia patients presented with microcephaly, dysmorphism and intellectual disability, similarly to our case (Cif et al., 2020). Other previously underreported features were also of note, such as early neonatal feeding issues, endocrinopathies, as well as intrauterine growth restriction, an unexplained feature also present in our patient’s clinical history. While most did display dystonic features, 17% of described patients with variants in *KMT2B* did not manifest movement disorders and may not develop dystonic features (Carecchio et al., 2019). In fact, Cif et al., 2020 identified nine patients harboring pathogenic KMT2B variants, in whom no dystonic features developed at time of assessment (median age 11.8 years with a range from 2.2–57.0 years). Additional features related to *KMT2B* variants have been documented such as failure to thrive, renal involvement, retinal dystrophy, oculomotor abnormalities like impaired saccades and strabismus, skin changes like cutis aplasia, and additional psychiatric comorbidities such as ADHD, anxiety, depression, and obsessive-compulsive disorder (Abela and Kurian, 2018; Ng et al., 2020). There have also been reports of myoclonus, seizures, spasticity, and sensorineural hearing loss (Meyer et al., 2017; Abela and Kurian, 2018; Zech et al., 2019; Owczarzak et al., 2022). Our patient presented with ADHD and seizures, two features reported in the literature. Regarding co-morbidities, KMT2B*-*related Dystonia has been associated with a risk of status dystonicus and endocrinopathies, such as hypothyroidism and precocious puberty (Meyer et al., 2017; Cao et al., 2020; Cif et al., 2020).

Within this large KMT2B-related disorder cohort investigated by Cif et al., 2020, only one case demonstrated the same frameshift variant as our 4-year-old patient (c.12_24dup (p.Ser9Glyfs*111). This variant was also *de novo* but was diagnosed by whole genome sequencing (WGS) for research purposes. This previously documented patient presented with unilateral lower limb dystonia with dysarthria, dysphagia, and retrocollis with age at onset of motor symptoms at 5 years old. Dystonia in this patient was refractory to different medical treatments (i.e., baclofen, carbamazepine, clonazepam, diazepam, intra-thecal baclofen, levodopa/carbidopa, and trihexyphenidyl), hence patient received deep brain stimulation insertion of the globus pallidus internus (GDi-DBS) at the age of 22 years; however, no longitudinal data were available regarding treatment.

GDi-DBS is being recognized as a highly beneficial therapeutic option in KMT2B-related Dystonia and provides a significant improvement in quality of life (Zech et al., 2019; Cif et al., 2020; Abel et al., 2021). In fact, a recent review of the literature demonstrated that, in most patients, a sustained response to deep brain stimulation, including restoration of independent ambulation, was observed (Zech et al., 2019). Similarly, Cif et al., 2020 showed that after 1-year post-DBS, >50% of patients showed significant (>30%) improvements of motor function and disability. Because KMT2B-related Dystonia appears to be refractory to commonly prescribed anti-dystonic agents, GDi-DBS could be prescribed as a first-line treatment, especially for patients diagnosed early. It is thus of utmost importance to ameliorate timely diagnosis for better management of patients and tailored treatment strategies. For our patient, EpiSign™ provided early diagnosis, and will permit close follow-up and monitoring for onset of dystonia symptoms. In the event of potential first signs of dystonia, earlier access to GDi-DBS will be possible and may significantly improve patient outcome and prognosis.

Timely diagnosis will permit young patients and their families to improve prognosis and obtain access to appropriate support networks. In our case, EpiSign™ testing has been invaluable in the early diagnosis of a KMT2B-related disorder in a 4-year-old patient with a *de novo* pathogenic *KMT2B* variant. If patient did not have access to this analysis, the next step for their diagnostic testing would have been a microcephaly panel based on her presentation at time of evaluation. This panel does not test *KMT2B*, and the patient would have likely remained unsolved. EpiSign™ uses proprietary machine learning-based algorithms to compare patients’ peripheral blood DNA methylation to defined episignatures within the EpiSign Knowledge Database (Levy et al., 2022). The EpiSign Knowledge Database is a clinical database with thousands of peripheral blood DNA methylation profiles including disorder-specific reference cohorts and population reference controls. EpiSign™ is the first clinically validated test designed to analyze genomic DNA methylation for diagnosis of genetic disorders and was first introduced in 2019; while it is currently a research test in Canada, it has recently begun to be adapted clinically in number of countries internationally (Sadikovic et al., 2021). Episignature analysis can also be useful for the reflex/second tier testing when genetic results are inconclusive, such as VUSs, providing strong functional evidence necessary for variant reclassification, however there has been utility demonstrated for use of EpiSign™ as a screening tool in first-tier testing. In a study assessing the first 207 patients clinically assessed by EpiSign™, 27.6% were positive for an episignature, with 35.3% (48/136) positivity for patients with previous ambiguous/inconclusive genetic findings (including VUS) and 11.3% positive rate in patients with clinical findings consistent with hereditary neurodevelopmental syndromes and no previous conclusive genetic findings (Sadikovic et al., 2021). In our case, EpiSign™ was particularly useful in providing an earlier diagnosis in a patient who would have received a large gene panel that did not include the causative gene (*KMT2B*) and would have then needed to wait for expanded clinical testing.

## 4 Methods

### 4.1 Episignature DNA methylation data analysis

Analysis of the DNA methylation array data was performed by the clinical bioinformatics laboratory (Verspeeten Clinical Genome Centre, London Health Sciences) using Illumina Infinium EPIC arrays. Methylation analysis was performed with the clinically validated EpiSign™ assay as previously described (Aref-Eshghi et al., 2019; Aref-Eshghi et al., 2020; Sadikovic et al., 2021; Kerkhof et al., 2022). Briefly, methylated and unmethylated signal intensity generated from the EPIC array was imported into R 3.5.1 for normalization, background correction, and filtering. Beta values ranging from 0 (no methylation) to 1 (complete methylation) were calculated as a measure of methylation level and processed through the established support vector machine (SVM) classification algorithm for EpiSign™ disorders. The EpiSign Knowledge Database composed of thousands of methylation profiles from reference disorder-specific and unaffected control cohorts was utilized by the classifier to generate disorder-specific methylation variant pathogenicity (MVP) scores. MVP scores are a measure of prediction confidence for each disorder, ranging from 0 (discordant) to 1 (highly concordant). For patients with full pathogenic variants, a positive EpiSign classification typically involves MVP scores greater than 0.5 in combination with concordant hierarchical clustering and multidimensional scaling. The MVP score is evaluated in combination with unsupervised clustering, generating hierarchical clustering and multidimensional scaling plots of the patient’s methylation data relative to the disorder specific EpiSign probe sets and population controls to determine if patient’s methylation profile is similar to an established episignature.

### 4.2 KMT2B single gene sequencing with deletion and duplication analysis

The KMT2B gene was evaluated for sequence anomalies and large deletions and/or duplications by a clinical accredited laboratory (Fulgent Genetics). Putative deletions/duplications identified by NGS are confirmed by an orthogonal method (qPCR or MLPA), unless exceeding an internally specified and validated quality score, beyond which deletions and duplications are considered real without further confirmation. Bioinformatics: The Fulgent Germline v2019.2 pipeline was used to analyze this specimen.

## Data Availability

The original contributions presented in the study are included in the article/Supplementary material, further inquiries can be directed to the corresponding authors.
